# Verknüpfung von Abrechnungsdaten gesetzlicher Krankenkassen mit Daten epidemiologischer Krebsregister: länderspezifische Möglichkeiten und Limitationen

**DOI:** 10.1007/s00103-021-03475-x

**Published:** 2021-12-23

**Authors:** Iris Pigeot, Brenda Bongaerts, Andrea Eberle, Alexander Katalinic, Joachim Kieschke, Sabine Luttmann, Martin Meyer, Alice Nennecke, Wolfgang Rathmann, Roland Stabenow, Heide Wilsdorf-Köhler, Bianca Kollhorst, Tammo Reinders

**Affiliations:** 1 Leibniz-Institut für Präventionsforschung und Epidemiologie – BIPS, Achterstr. 30, 28359 Bremen, Deutschland; 2 Fachbereich Mathematik und Informatik, Universität Bremen, Bremen, Deutschland; 3 Deutsches Diabetes-Zentrum (DDZ), Leibniz-Zentrum für Diabetes-Forschung, Heinrich-Heine-Universität, Düsseldorf, Deutschland; 4 Landeskrebsregister Bremen, Leibniz-Institut für Präventionsforschung und Epidemiologie – BIPS, Bremen, Deutschland; 5 Institut für Sozialmedizin und Epidemiologie, Universität zu Lübeck, Lübeck, Deutschland; 6 Registerstelle, Epidemiologisches Krebsregister Niedersachsen, Oldenburg, Deutschland; 7 Bayerisches Landesamt für Gesundheit und Lebensmittelsicherheit, Nürnberg, Deutschland; 8 Freie und Hansestadt Hamburg, Behörde für Wissenschaft, Forschung, Gleichstellung und Bezirke, Hamburgisches Krebsregister, Hamburg, Deutschland; 9 Gemeinsames Krebsregister der Länder Berlin, Brandenburg, Mecklenburg-Vorpommern, Sachsen-Anhalt und der Freistaaten Sachsen und Thüringen, Berlin, Deutschland

**Keywords:** Direktes Linkage, FAIR-Prinzipien, GePaRD, Indirektes Linkage, Record Linkage, Direct linkage, FAIR principles, GePaRD, Indirect linkage, Record linkage

## Abstract

**Hintergrund:**

In den letzten Jahren wird verstärkt gefordert, Forschungsdaten gemäß den sog. FAIR-Prinzipien für eine Nachnutzung aufzubereiten. Dadurch könnten zukünftige Projekte auf einer breiteren Datengrundlage durchgeführt sowie durch Verknüpfung verschiedener Datenquellen neue Fragestellungen untersucht werden.

**Fragestellung:**

Eruiert werden soll, inwieweit Abrechnungsdaten gesetzlicher Krankenversicherungen mit den Daten der Landeskrebsregister (LKR) überregional verknüpft werden können, um die in den Abrechnungsdaten fehlenden Informationen zu Krebserkrankungen ergänzen und die Validität der dortigen Angaben zur Tumordiagnose beurteilen zu können. Der Fokus liegt dabei auf der Beschreibung der länderspezifischen Anforderungen für einen solchen Datenabgleich.

**Material und Methoden:**

Als Datenquellen wurden die Pharmakoepidemiologische Forschungsdatenbank GePaRD des Leibniz-Instituts für Präventionsforschung und Epidemiologie – BIPS und sechs Krebsregister herangezogen. Zur Verknüpfung wurden vergleichend das logistisch aufwendige direkte Linkage- und ein weniger aufwendiges indirektes Linkage-Verfahren angewandt. Dazu mussten für GePaRD und für jedes LKR die Genehmigungen der jeweils zuständigen Behörde eingeholt werden.

**Ergebnisse:**

Hinsichtlich der Verknüpfung von LKR-Daten mit GePaRD zeigten sich gravierende Unterschiede in der Datenbereitstellung (vollständige Ablehnung bis hin zu einer unkomplizierten Umsetzung).

**Diskussion:**

In Deutschland müssen einheitliche Rahmenbedingungen geschaffen werden, um eine angemessene Nachnutzung und eine Verknüpfung von personenbezogenen Gesundheitsdaten zu Forschungszwecken im Sinne der FAIR-Prinzipien zu ermöglichen. Bezüglich der Verknüpfung von LKR-Daten mit anderen Datenquellen könnte das neue Gesetz zur Zusammenführung von Krebsregisterdaten Abhilfe schaffen.

## Abstract (en)

**Background:**

In recent years, there has been an increasing demand for the reuse of research data in accordance with the so-called FAIR principles. This would allow researchers to conduct projects on a broader data basis and to investigate new research questions by linking different data sources.

**Objectives:**

We explored if nationwide linking of claims data from statutory health insurances (SHI) with data from population-based cancer registries can be used to obtain additional information on cancer that is missing in claims data and to assess the validity of SHI tumour diagnoses. This paper focuses on describing the specific requirements of German federal states for such data linkage**.**

**Materials and methods:**

The Pharmacoepidemiological Research Database GePaRD at the Leibniz Institute for Prevention Research and Epidemiology – BIPS and six cancer registries were used as data sources. The logistically complex direct linkage was compared with a less complex indirect linkage. For this purpose, permission had to be obtained for GePaRD and for each cancer registry from the respective responsible authority.

**Results:**

Regarding the linkage of cancer registry data with GePaRD, the cancer registries showed profound differences in the modalities for data provision, ranging from a complete rejection to an uncomplicated implementation of linkage procedures.

**Discussion:**

In Germany, a consistent legal framework is needed to adequately enable the reuse and record linkage of personal health data for research purposes according to the FAIR principles. The new law on the consolidation of cancer registry data could provide a remedy regarding the linkage of cancer registry data with other data sources.

## Einleitung

Qualitätsgesicherte Primärdaten sind eine wesentliche Grundlage für wissenschaftliche Erkenntnisse in nahezu allen Wissenschaftsbereichen. Aber auch routinemäßig erhobene Daten, sog. Sekundärdaten, können bei der Beantwortung von Forschungsfragen einen relevanten Beitrag leisten. Für die Versorgungsforschung sind Sekundärdaten sogar essenziell, auch wenn sie nicht primär zu Forschungszwecken erhoben wurden. Um das Potenzial sowohl von Primär- als auch von Sekundärdaten vollständig und im Sinne einer effizienten Ressourcennutzung ausschöpfen zu können, forderte die Organisation für wirtschaftliche Zusammenarbeit und Entwicklung (OECD) bereits 2007 [1] einen einfachen Zugang zu sämtlichen Forschungsdaten für die gesamte wissenschaftliche Gemeinschaft. Dabei lassen sich auch zu Forschungszwecken genutzte Sekundärdaten unter dem Begriff „Forschungsdaten“ subsumieren. In den letzten Jahren wird verstärkt gefordert [2, 3], Forschungsdaten zu veröffentlichen und für eine Nutzung durch externe Forschende zur Verfügung zu stellen (*Data Sharing*). Dazu müssen die Daten entsprechend den sog. FAIR-Prinzipien [4] aufbereitet werden: Sie sollen danach auch für Dritte jederzeit auffindbar („findable“), zugänglich („accessible“), interoperabel („interoperable“) und wiederverwendbar („reusable“) sein. Dadurch bietet sich insbesondere die Möglichkeit, die eigene Forschung durch die Auswertung externer Datenquellen auf einer breiteren Datengrundlage durchzuführen sowie durch die Verknüpfung verschiedener externer Datenquellen mit den eigenen Daten neuartige Fragestellungen zu entwickeln und zu untersuchen. Zudem können durch die Verknüpfung verschiedener Datenquellen zum einen die jeweiligen Inhalte gegenseitig um fehlende Inhalte ergänzt und zum anderen vorhandene Informationen wechselseitig validiert werden. In Deutschland wird diesem Bestreben einer „FAIRifizierung“ von Daten durch die Initiative des Aufbaus einer Nationalen Forschungsdateninfrastruktur (NFDI; [5, 6]) Rechnung getragen.

Allerdings erweist sich dies als schwierig, da in Deutschland zurzeit in verschiedenen Datenbanken noch keine einheitliche, die jeweilige Person eindeutig identifizierende Nummer verwendet wird, die ein direktes Linkage (d. h. eine individuelle Verknüpfung von Daten) unkompliziert ermöglichen würde (s. dazu auch [7]). Auch wenn die Europäische Datenschutzgrundverordnung (EU-DSGVO) einen allgemeinen Standard bzgl. des Datenschutzes für Europa festlegt, gibt es bei der Umsetzung in den Mitgliedsstaaten große Variationen. In Deutschland unterliegen personenbezogene Daten einem hohen Datenschutz. Vor allem Gesundheitsdaten, wie die sensiblen Daten in Krankheitsregistern, sowie Sozialdaten, wie etwa die Abrechnungsdaten der gesetzlichen Krankenversicherungen (GKV), werden durch eine strikte Gesetzgebung im Rahmen der EU-DSGVO besonders geschützt.

In einem von der Deutschen Forschungsgemeinschaft geförderten Projekt sollte anhand einer konkreten Fragestellung untersucht werden, ob GKV-Abrechnungsdaten mit den Daten der Landeskrebsregister (LKR) verknüpft werden können, um die in den Abrechnungsdaten fehlenden Informationen über Krebserkrankungen, wie z. B. das Tumorstadium, zu ergänzen und eine Aussage über die Validität der Tumordiagnose (Tumorpathologie) in den GKV-Daten treffen zu können. Eine solche Verknüpfung der Abrechnungsdaten mit den Daten eines Krebsregisters würde somit einen essenziellen Beitrag z. B. zur Untersuchung der möglichen Modifikation eines Krebsrisikos durch Medikamente leisten. In unserem Projekt wurde insbesondere der Frage nachgegangen, ob das logistisch sehr aufwendige Linkage mittels einer eindeutigen Kennung, im Folgenden als direktes Linkage bezeichnet, ohne nennenswerten Qualitätsverlust durch ein sog. indirektes Linkage nur basierend auf Informationen, die in beiden Datenquellen vorhanden sind, ersetzt werden kann. Zu diesem Zweck muss für jedes LKR die Genehmigung für beide Linkage-Ansätze eingeholt werden.

Der Fokus dieses Artikels liegt auf der Beschreibung der länderspezifischen Anforderungen für eine solche Verknüpfung von Datenquellen. Es werden daher im Folgenden zunächst die zu verknüpfenden Datenquellen kurz vorgestellt, bevor das direkte und das indirekte Linkage beschrieben werden. Im anschließenden Abschnitt werden die länderspezifischen Regelungen bei der Umsetzung der Verknüpfung von Krebsregisterdaten mit anderen Datenquellen dargestellt. Dabei wird auf die besondere Herausforderung eingegangen, dass die notwendigen Bestimmungen, einschließlich datenschutzrechtlicher Regelungen, für Einrichtung und Betrieb der flächendeckenden Krebsregistrierung nach § 65c Fünftes Buch Sozialgesetzbuch (SGB V) dem Landesrecht vorbehalten sind. Das heißt, Krebsregisterdaten werden mit einer landesgesetzlich festgelegten Einschränkung des Rechts auf informationelle Selbstbestimmung erhoben, woraus sich eine eng begrenzte Zweckbindung ergibt. Die Ausgestaltung dieser Zweckbindung führt für jedes LKR zu unterschiedlichen Modalitäten zur Datenbereitstellung für Forschungsanfragen. In der abschließenden Diskussion wird noch einmal auf die Notwendigkeit einer bundesweiten Regelung eingegangen und die bereits erfolgten Schritte auf diesem Weg werden dargestellt.

## Beschreibung der Datenquellen und ihrer Pseudonymisierung

### Pharmakoepidemiologische Forschungsdatenbank GePaRD

Das Leibniz-Institut für Präventionsforschung und Epidemiologie – BIPS trägt die wissenschaftliche Verantwortung für die Pharmakoepidemiologische Forschungsdatenbank (*German Pharmacoepidemiological Research Database*, GePaRD; [8]), die aktuell Abrechnungsdaten von 4 GKV der Jahre 2004–2018 umfasst. Die Datenbank enthält Informationen von ca. 25 Mio. gesetzlich Krankenversicherten aus allen Regionen Deutschlands. Für jede versicherte Person sind sowohl demografische Angaben wie Geschlecht, Geburtsjahr und der Amtliche Gemeindeschlüssel (5-stellig) als auch Angaben zu Krankenhausaufenthalten, ambulanten Arztbesuchen und Verschreibungen enthalten. Die Krankenhausdaten liefern Informationen über das Datum des Aufenthalts, Diagnosen (codiert nach ICD-10-GM, *German Modification*), Gründe für Aufnahme und Entlassung aus dem Krankenhaus und therapeutische und diagnostische Prozeduren. Abrechnungsdaten der ambulanten Arztbesuche beinhalten Prozeduren und Diagnosen (kodiert nach ICD-10-GM).

Der Zugang zu GePaRD ist gemäß § 75 SGB X nur nach vorheriger Genehmigung der beteiligten Krankenkassen und der jeweiligen Aufsichtsbehörden und jeweils nur für die genehmigte Fragestellung möglich. Der Zugang zu GePaRD ist unter Einhaltung des üblichen Genehmigungsverfahrens und der datenschutzrechtlichen Vorgaben grundsätzlich auch für Gastwissenschaftlerinnen und -wissenschaftler, die vertraglich an das BIPS angeschlossen sind, möglich. Lediglich zwei der vier an GePaRD beteiligten Krankenversicherungen erklärten ihre grundsätzliche Bereitschaft zur Projektteilnahme, sodass für dieses Projekt nur deren Daten genutzt werden konnten. Da es sich um personenbezogene Sozialdaten handelt, wird die Datenbank von einer Vertrauensstelle geführt, die räumlich, personell und organisatorisch vom BIPS getrennt ist und die Versichertenkennzeichen, Arztnummern (seit 01. 07. 2008 lebenslange Arzt- und Betriebsstättennummern), Institutionskennzeichen der Apotheken sowie Institutionskennzeichen der Krankenhäuser pseudonymisiert. Dazu werden die vorhandenen Identifikationsvariablen durch fortlaufende Nummern ersetzt. Die Zuordnung der ursprünglichen Identifikationsnummer zur neu vergebenen projektinternen Identifikationsnummer wird in Zuordnungstabellen dokumentiert. Für jede pseudonymisierte Variable wird eine eigene Zuordnungstabelle geführt. Die Zuordnung erfolgt in einer willkürlichen Reihenfolge; unmittelbare Rückschlüsse aus der projektinternen Identifikationsnummer auf die ursprüngliche Identifikation sind damit ausgeschlossen.

Abrechnungsdaten gesetzlicher Krankenkassen sind ein wichtiger Bestandteil epidemiologischer Studien in der Versorgungs- und Arzneimittelrisikoforschung. Die Vorteile dieser Daten liegen in den weitestgehend vollständigen Angaben auf Individualebene, in der großen Zahl an Patientinnen und Patienten, wodurch z. B. die Untersuchung sehr seltener Arzneimittelrisiken möglich ist, und in dem Datenumfang über viele Jahre, der es z. B. ermöglicht, auch sich erst später manifestierende Arzneimittelrisiken zu untersuchen. Auch sind Informationen zum Arzneimittelgebrauch zuverlässiger als in Feldstudien erhobene Informationen, da Ärztinnen und Ärzte und Patientinnen und Patienten nicht von der zu untersuchenden Fragestellung beeinflusst werden [9]. Zudem können auch besondere Patientengruppen, wie z. B. alte Personen, Pflegebedürftige oder Schwangere, in Studien berücksichtigt werden. Die in GePaRD enthaltenen Angaben werden direkt von den Krankenkassen übermittelt, sodass keine studienbedingten Verzerrungen, wie z. B. Erinnerungsfehler, auftreten. Da die Daten zu Abrechnungszwecken und nicht primär für die Forschung erhoben werden, fehlen jedoch z. T. spezifische Informationen über Erkrankungen, wie z. B. das Tumorstadium.

### Landeskrebsregister (LKR)

Die Registrierung von Krebserkrankungen wird in Deutschland auf Bundes- und Länderebene geregelt, dabei werden die Rahmenbedingungen durch Bundesgesetze vorgegeben. Für dieses Projekt wurden die LKR von Bayern, Bremen, Hamburg, Niedersachsen, Schleswig-Holstein und das Gemeinsame Krebsregister der Länder Berlin, Brandenburg, Mecklenburg-Vorpommern, Sachsen-Anhalt und der Freistaaten Sachsen und Thüringen (GKR) zum einen aufgrund der regionalen Verteilung der Versicherten in GePaRD einbezogen. Zum anderen hatten diese bei einer ersten Anfrage ihre grundsätzliche Bereitschaft zur Teilnahme an dem Projekt erklärt. Die LKR der Bundesländer sind populations- und behandlungsortbezogen und enthalten demografische Angaben wie Geschlecht, Geburtsjahr und den Amtlichen Gemeindeschlüssel. Die Daten zu den Krebserkrankungen umfassen die Tumordiagnose (codiert nach ICD-10-WHO, Weltgesundheitsorganisation) mit zugehörigem Datum, Lokalisation und Histologie des Tumors, Stadium der Erkrankung, Art der Diagnosesicherung, Art der Therapie und Todesursache mit Angabe des Monats und Jahres. Nach Inkrafttreten des Krebsfrüherkennungs- und -registergesetzes (KFRG; [10]) im Jahr 2013 wurden die meisten vormals rein bevölkerungsbezogenen Krebsregister zu klinisch-epidemiologischen Registern ausgebaut, die nun auch detaillierte Daten zu Verlauf und Therapie der Erkrankung zu allen im Einzugsgebiet behandelten Patientinnen und Patienten erfassen. Die Daten der LKR entstammen verschiedenen Quellen wie Meldungen über Neuerkrankungen auf Basis eines ärztlichen Behandlungsverhältnisses, Meldungen aus der pathologischen Diagnostik oder Auswertungen von Informationen aus Todesbescheinigungen, auch Death-Certificate-only-(DCO-)Fälle genannt. Die verfügbaren Diagnosejahre unterscheiden sich zwischen den Bundesländern (Tab. 1).

**Table 1:** 

Register	Diagnosejahre
Bayern [11]	2002–2019
Bremen [12]	1998–2019
Gemeinsames Krebsregister der Länder Berlin, Brandenburg, Mecklenburg-Vorpommern, Sachsen-Anhalt und der Freistaaten Sachsen und Thüringen [13]	1961–2015
Hamburg [14]	1990–2018
Niedersachsen [15]	2001–2019
Schleswig-Holstein [16]	1999–2017

Der Zugang zu den personenbezogenen Daten der Krebsregister ist auch für die wissenschaftliche Nutzung möglich und muss bei jedem Register einzeln beantragt werden (s. dazu auch den Abschnitt „Länderspezifische Umsetzung des Datenlinkage“ und Tab. 2). Im Rahmen der Datenspeicherung werden in den Vertrauensstellen der Krebsregister, die getrennt von den Registerstellen sind, sog. Kontrollnummern generiert, die eine pseudonymisierte Datenhaltung ermöglichen. Hierbei kommen zwei unterschiedliche Chiffrierungsmethoden zur Anwendung. In einem ersten Schritt erfolgt eine deterministische Einwegverschlüsselung (MD5-Einwegverschlüsselung), die standardisierte Identifizierungsmerkmale so verschlüsselt, dass keine Klartextangaben mehr erkennbar sind und somit keine einzelnen Personen mehr identifiziert werden können. Jedes Merkmal wird auf eine Zeichenkette von 16 Zeichen abgebildet. Um einer Rückverschlüsselung vorzubeugen, wird in einem zweiten Schritt zusätzlich eine symmetrische Verschlüsselung nach dem sog. International Data Encryption Algorithm (IDEA) durchgeführt. Dieses komplexe Verfahren resultiert in 22 jeweils 23-stelligen Kontrollnummern pro Person, die den akademischen Titel, Vor- und Nachnamen, Geburtsnamen, frühere Namen und den Tag des Geburtsdatums verschlüsseln. Im Krebsregister wird die IDEA-Verschlüsselung mittels eines krebsregisterspezifischen Schlüssels vorgenommen. Für einen Datenabgleich mit anderen externen Stellen, wie hier vorgesehen, sind spezifische projektbezogene IDEA-Abgleichschlüssel (das sog. Austauschformat) erforderlich, d. h. die Verschlüsselung mit dem krebsregisterspezifischen Schlüssel wird rückgängig gemacht und es wird mit dem projektspezifischen Schlüssel neu verschlüsselt. Damit erhält ein und dieselbe Person in unterschiedlichen Projekten unterschiedliche Kontrollnummern. Die Registerstellen haben ausschließlich Zugriff auf die pseudonymisierten Daten. Beim Zentrum für Krebsregisterdaten (ZfKD) können ausschließlich anonymisierte bundesweite Daten für Forschungszwecke beantragt werden, die dementsprechend nicht zur Verknüpfung mit anderen Datenbanken verwendet werden können, sodass für diese Studie die einzelnen LKR kontaktiert werden mussten.

**Table 2:** 

Krebsregister	Einzureichende Unterlagen	Entscheidung (gefällt durch zum Zeitpunkt der Entscheidung zuständige Stelle)
Bayern	Antragsformular	Datenübermittlung zugestimmt (Beirat des Bayerischen Krebsregisters)
	Studienprotokoll (bzw. Exposé), inkl. Datenflussbeschreibung	
	DSK GePaRD/DSK DFG-Linkage	
	Genehmigung für das DFG-Projekt durch Aufsichtsbehörden der Krankenkassen (Bremer Senatorin und BVA)	
Bremen	Antrag auf Datennutzung	Datenübermittlung nach Berücksichtigung des Votums des wissenschaftlichen Beirats zugestimmt (Bremer Senatorin für Wissenschaft, Gesundheit und Verbraucherschutz)
	Studienprotokoll (bzw. Exposé), inkl. Datenflussbeschreibung	
	DSK GePaRD	
Gemeinsames Krebsregister der Länder Berlin, Brandenburg, Mecklenburg-Vorpommern, Sachsen-Anhalt und der Freistaaten Sachsen und Thüringen	Datennutzungsantrag, inkl. Studienprotokoll und Datenflussbeschreibung	Datenübermittlung abgelehnt
Hamburg	Hamburgspezifisches DSK, inkl. Datenflussbeschreibung (erweitertes Studienprotokoll)	Datenübermittlung zugestimmt (Hamburger Behörde für Wissenschaft, Forschung, Gleichstellung und Bezirke (zustimmende Stellungnahme von Hamburgischem Beauftragten für Datenschutz und Informationsfreiheit sowie der Ärztekammer Hamburg))
	Formblatt gemäß § 9 Abs. 1–6 HmbKrebsRG	
	DSK GePaRD	
Niedersachsen	Studienprotokoll (bzw. Exposé), inkl. Datenflussbeschreibung und Fallzahlschätzung	Datenübermittlung zugestimmt (Niedersächsisches Ministerium für Soziales, Gesundheit und Gleichstellung)
	DSK GePaRD/DSK DFG-Linkage	
	Genehmigung für das DFG-Projekt durch Aufsichtsbehörden der Krankenkassen (Bremer Senatorin und BVA)	
Schleswig-Holstein	Studienprotokoll (bzw. Exposé), inkl. Datenflussbeschreibung	Datenübermittlung abgelehnt (Ministerium für Soziales, Gesundheit, Jugend, Familie und Senioren des Landes Schleswig-Holstein)
	DSK GePaRD/DSK DFG-Linkage	
	Genehmigung für das DFG-Projekt durch Aufsichtsbehörden der Krankenkassen (Bremer Senatorin und BVA)	
	Stellungnahme Ethikkommission	

*BVA* Bundesversicherungsamt (jetzt: Bundesamt für Soziale Sicherung), *DFG* Deutsche Forschungsgemeinschaft, *DFG-Linkage* DFG-Projekt „Evaluierung eines indirekten Linkage-Ansatzes anhand einer Beispielstudie zum Risiko einer Krebsneuerkrankung und der Krebsmortalität bei Patienten und Patientinnen mit Typ-2-Diabetes unter Behandlung mit verschiedenen Antidiabetika“, *DSK* Datenschutzkonzept, *GePaRD* Pharmakoepidemiologische Forschungsdatenbank (German Pharmacoepidemiological Research Database), *HmbKrebsRG* Hamburgisches Krebsregistergesetz

## Record Linkage-Verfahren

Aufgrund der datenschutzrechtlichen Anforderungen und des administrativen Aufwands gibt es nur wenige Studien in Deutschland, die verschiedene Forschungsdatenrepositorien mit Krebsregisterdaten für die epidemiologische Forschung verknüpft haben. Ein Beispiel ist ein Record Linkage von Daten aus einem Disease-Management-Programm für Patientinnen und Patienten mit Diabetes mellitus Typ 2 mit den Daten des Krebsregisters Nordrhein-Westfalen im Bezirk Münster [17]. Eine deutschlandweite Verknüpfung von Daten verschiedener gesetzlicher Krankenkassen mit den Daten der Krebsregister hat in dieser Form noch nicht stattgefunden.

Auch sonst steht die Forschung zur Verknüpfung von Primär‑, Sekundär- und Registerdaten in Deutschland im Vergleich zu anderen europäischen Ländern, wie etwa Dänemark [18], noch am Anfang (s. z. B. [19–21]). In Kooperation verschiedener Fachgesellschaften wurde 2019 die Gute Praxis Datenlinkage (GPD) erarbeitet und publiziert [22].

Allgemein kann eine direkte Verknüpfung zweier Datenquellen deterministisch über ein eindeutiges personenbezogenes Zuordnungsmerkmal wie die Krankenversicherungsnummer oder eine Personenkennnummer erfolgen. Dabei ist es nicht erforderlich, dass diese Angaben im Klartext vorliegen. Eine weitere Möglichkeit ist eine probabilistische Verknüpfung mittels definierter Übereinstimmungswahrscheinlichkeiten der verwendeten personenbezogenen Merkmale. Dieser Ansatz wird verwendet, wenn die vorliegenden personenbezogenen Merkmale, wie z. B. Name, Vorname, keine eindeutige Zuordnung zulassen. Die Kombination mit weiteren Merkmalen ist möglich. Aus Datenschutzgründen kann auch hier eine Überverschlüsselung, also eine nochmalige Verschlüsselung der bereits verschlüsselten Information, gewählt werden; in den Krebsregistern erfolgt dies routinemäßig über Kontrollnummern.

Da man in beiden Fällen, der deterministischen sowie der probabilistischen Verknüpfung, personenidentifizierende Merkmale benötigt, besteht eine Alternative darin, ein indirektes Linkage über nicht-personenidentifizierende Merkmale durchzuführen, die in beiden Datenquellen vorhanden sind. Hierfür könnten in der hier interessierenden Fallstudie z. B. Angaben zum Datum der Tumordiagnose genutzt werden. Die Vorteile des Linkage über nicht-personenidentifizierende Merkmale ergeben sich direkt aus den Nachteilen des Linkage über personenidentifizierende Merkmale (hohe datenschutzrechtliche Anforderungen, hoher administrativer Aufwand). Allerdings muss beim indirekten Linkage in Kauf genommen werden, dass dieses Verfahren zu falsch verknüpften Daten oder zu einer Nichtverknüpfung von eigentlich zusammengehörenden Datensätzen führen kann.

### Direktes Linkage

Ein direktes Linkage zwischen den Daten der LKR und anderen Datenquellen über pseudonymisierte Identitätsdaten wird in den Krebsregistern routinemäßig über Kontrollnummern durchgeführt, beispielsweise beim Abgleich von Daten des Mammographie-Screening-Programms mit bevölkerungsbezogenen Krebsregistern [23, 24]. In der Vertrauensstelle des jeweiligen Krebsregisters werden dazu Kontrollnummern aus den Feldern „Titel“, „Name“, „Vorname“, „Geburtsname“, „früherer Name“ und „Tag des Geburtsdatums“ sowie die im Klartext vorliegenden Merkmale „Geschlecht“, „Geburtsmonat“, „Geburtsjahr“ und „Amtlicher Gemeindeschlüssel“ als Verknüpfungsschlüssel gebildet. Da in GePaRD die zur Erzeugung der Kontrollnummern notwendigen Informationen nicht alle vorhanden sind, mussten für das direkte Record Linkage mit Krebsregisterdaten die zu verlinkenden Versicherten aus GePaRD zunächst ausgewählt werden. Dazu wurde eine Kohorte von Diabetikerinnen und Diabetikern mit einem Wohnsitz in Bayern, Bremen, Hamburg oder Niedersachsen aufgesetzt, die zwischen 2004 und 2015 eine Diabetesbehandlung begonnen haben, d. h., es wurden alle Versicherten mit mindestens einer Abgabe eines glukosesenkenden Präparats oder von Insulin (ATC-Code A10) zwischen 2004 und 2015 ausgewählt, die durchgängig für mindestens 365 Tage versichert und bei der Erstverschreibung mindestens 18 Jahre alt waren. Zudem mussten die Versicherten mindestens eine Diagnose von Typ-2-Diabetes (ICD-10-GM E11, E14) aufweisen, jedoch durften sie keine Typ-1-Diabetes oder eine andere Diabetesdiagnose aufweisen, um in die Kohorte aufgenommen zu werden.

Anschließend wurden die so identifizierten Versicherten zusammen mit einer projektspezifischen Identifikationsnummer (Projekt-ID) über eine Vertrauensstelle an die teilnehmenden Krankenkassen geschickt. Dort wurden dann die Kontrollnummern generiert und unverschlüsselte Informationen zu Geschlecht, Wohnort, Monat und Jahr des Geburtsdatums hinzugefügt. Im Anschluss übermittelte die Vertrauensstelle die Versicherten mit der Projekt-ID, den Kontrollnummern und den unverschlüsselten Informationen gemäß ihrem Wohnort im Jahr 2017 an das jeweilige Krebsregister. Im Rahmen des automatisierten Abgleichs erfolgte, vereinfacht dargestellt, die Bestimmung eines Übereinstimmungsgewichts anhand der Übereinstimmungswahrscheinlichkeiten der Einzelmerkmale für jedes Datenzeilenpaar. Zur Bewertung der Übereinstimmungsgewichte wurden zwei Schranken festgelegt: Die untere Schranke gibt die Schwelle an, unterhalb der Übereinstimmungen der Datenzeilen ausgeschlossen werden. Die obere Schranke legt die Grenze fest, ab der von sicheren Übereinstimmungen ausgegangen wird. Die Entscheidung über die Zusammengehörigkeit der Datenzeilen in der dazwischen liegenden Grauzone erfolgte manuell in dem jeweiligen Krebsregister. Die so identifizierten Paare wurden anschließend zusammen mit der Projekt-ID, der Tumordiagnose und dem Diagnosedatum, dem Tumorstadium, dem Vitalstatus und der Todesursache an GePaRD übermittelt. Da für die Evaluierung des im folgenden Abschnitt beschriebenen und weniger aufwendigen indirekten Linkage-Ansatzes exemplarisch der Fokus in diesem Projekt auf Darmkrebs (ICD-10-WHO C18–C21) und Schilddrüsenkrebs (ICD-10-WHO C73) gelegt wurde, haben die Krebsregister alle entsprechenden Fälle zwischen 2004 und 2015 extrahiert und die Projekt-ID für diejenigen Krebsfälle hinzugefügt, für die es beim direkten Linkage über Kontrollnummern eine Übereinstimmung mit einem/r Versicherten aus GePaRD gab.

### Indirektes Linkage

Für das indirekte deterministische Linkage wurde die in beiden Datenquellen vorhandene Information herangezogen. Diese beinhaltete das Geburtsjahr, Geschlecht, Wohnort im Jahr 2017 (GePaRD) bzw. Wohnort bei Diagnosestellung (LKR) und Krebsart (Darm oder Schilddrüse). Zusätzlich durften sich die Datumsangaben bzgl. der Krebsdiagnose in den beiden Datenquellen nur um höchstens 90 Tage unterscheiden. Dabei wurde in GePaRD das Aufnahmedatum ins Krankenhaus bei der ersten Diagnose oder das erste Datum einer Gebührenordnungsposition, die mit der Krebsdiagnose verknüpft war, verwendet, während im Krebsregister das Datum der Erstmeldung genutzt wird, d. h. es wurden nur inzidente Fälle berücksichtigt. Im Fall, dass mehr als eine Übereinstimmung gefunden wurde, wurden hierarchisch weitere Kriterien angesetzt, um eine eindeutige Zuordnung zu erhalten. So wurden bevorzugt Paare ausgewählt, bei denen der ICD-Code auf vier Stellen genau übereinstimmte. Falls es keine Paare mit einer vierstelligen ICD-Code-Übereinstimmung gab, wurden als nächstes Paare ausgewählt, bei denen der ICD-Code auf drei Stellen genau übereinstimmte. Darüber hinaus wurden in GePaRD prioritär Krebsfälle, die im Krankenhaus diagnostiziert wurden, vor Krebsfällen aus dem ambulanten Bereich ausgewählt. Zudem wurde für die Identifikation von Paaren der kleinste zeitliche Abstand zwischen dem Diagnosedatum in beiden Datenquellen gewählt. Wenn dann immer noch nicht zwischen zwei oder mehr möglichen Treffern unterschieden werden konnte, wurde ein Paar zufällig ausgewählt, sodass aus jeder Datenquelle jede Person nur einmal verlinkt wurde. Die Ergebnisse des indirekten Linkage werden in einem separaten Paper dargestellt.

## Länderspezifische Umsetzung des Datenlinkage

In Deutschland ist die Arbeitsweise der LKR entsprechend § 65c Abs. 1 S. 7 SGB V in den Landesgesetzen geregelt. Die grundsätzlichen Rahmenbedingungen sind durch verschiedene Bundesgesetze (Bundeskrebsregisterdatengesetz von 2009 [25], KFRG von 2013 [8] bzw. § 65c SGB V (zuletzt geändert durch Art. 4 G v. 12.05.2021)) vorgegeben, die im Detail unterschiedlich in den Landesgesetzen umgesetzt wurden.

Das Kontrollnummernverfahren wurde bereits beim Aufbau der Krebsregistrierung in Deutschland Mitte der 1990er-Jahre entwickelt, um in erster Linie die sensiblen Daten bei der Verarbeitung zu schützen und Meldungen zu einem Erkrankungsfall und einer Person sicher zusammenzuführen. Die Nutzung der Kontrollnummer als Instrument zur Verknüpfung der Krebsregisterdaten mit anderen Datenquellen ist erst in den letzten Jahren zunehmend in den Fokus gerückt.

Da in dem oben beschriebenen Projekt die Evaluierung des indirekten Linkage im Vergleich zum direkten Linkage von besonderem Interesse war, wurde die Umsetzung beider Verfahren bei den jeweiligen LKR gleichzeitig beantragt. Dementsprechend unterscheidet Tab. 2 in dem Überblick über die Art der Antragstellung, die einzureichenden Unterlagen und die jeweils erfolgte Entscheidung nicht nach dem Typ des Verfahrens.

Wie man der Tabelle entnehmen kann, führten die verschiedenen Landesgesetze zu formal variierenden Antragsverfahren und zu teils unterschiedlichen Einschätzungen der Aufsichtsbehörden und damit zu unterschiedlichen Ergebnissen des Antragsverfahrens. Nachfolgend sind die jeweiligen Antragsverfahren bei den beteiligten Krebsregistern kurz dargestellt.

### Bayern.

Das Antragsverfahren in Bayern gestaltete sich über ein entsprechendes Antragsformular unproblematisch. Einer pseudonymen Datennutzung durch Dritte gemäß Art. 13 des Bayerischen Krebsregistergesetzes (BayKRegG) wurde zugestimmt, da im weiteren Datenfluss des Projekts keine Reidentifizierungsrisiken gesehen wurden.

### Bremen.

In Bremen ist für Datenbereitstellungen eine behördliche Genehmigung notwendig. Nach eingehender Prüfung des Antrags durch die zuständige Behörde und nach Klärung der Datenflüsse mit den Antragstellenden wurde gemäß den landesrechtlichen Bestimmungen das Votum des wissenschaftlichen Beirats eingeholt. Im Anschluss erfolgt die Genehmigung durch die zuständige Behörde.

### Gemeinsames Krebsregister der Länder Berlin, Brandenburg, Mecklenburg-Vorpommern, Sachsen-Anhalt und der Freistaaten Sachsen und Thüringen (GKR).

Das GKR arbeitet im Unterschied zu den meisten anderen LKR als länderübergreifendes und ausschließlich epidemiologisches Krebsregister. In der Datennutzungsvereinbarung des GKR ist festgehalten, dass Einzelfalldaten nicht deanonymisiert und nicht mit weiteren Daten auf Individualebene verlinkt werden dürfen. Kenngrößen dürfen lediglich auf aggregiertem Niveau den Daten zugespielt werden. Es wurde dennoch für das Projekt eine vom herkömmlichen Vorgehen des GKR abweichende Lösung angestrebt, für die im Einklang mit den gesetzlichen Grundlagen ein Konzept erarbeitet und dem Datenschutzbeauftragten zur Prüfung vorgelegt werden sollte. Das GKR musste den Antrag auf Daten-Linkage jedoch ablehnen, da zwar ein neues Anonymisierungskonzept ausgearbeitet wurde, dieses jedoch nicht zeitnah vom Berliner Beauftragten für Datenschutz und Informationsfreiheit (BlnBDI) beurteilt werden konnte. Darüber hinaus hat die Einführung einer neuen Software den Routinebetrieb behindert.

### Hamburg.

Zu derselben Einschätzung wie das Bremer Krebsregister gelangte auch das Hamburger Krebsregister (HKR). Zurzeit ist im HKR ein Kohortenabgleich nur mit Einwilligung der betroffenen Personen möglich, da es für die Datenverarbeitung von Personen ohne Krebserkrankung im Register mit Ausnahme von Daten von Teilnehmerinnen am Mammographie-Screening keine Rechtsgrundlage gibt. Um diesem Problem zu begegnen, wurde extra für dieses Projekt ein Studienzentrum in Hamburg räumlich getrennt vom HKR eingerichtet, in dem die Daten durch einen Mitarbeiter des BIPS direkt verlinkt werden konnten.

### Niedersachsen.

Durch die Vollerfassung aller Fälle durch das Krebsregister wurde befürchtet, dass durch das indirekte Linkage eine Person, die mit ihren Eigenschaften bzgl. Alter, Geschlecht, Amtlichen Gemeindeschlüssels und Diagnose einzigartig ist, reidentifiziert werden könnte (s. auch § 11 Abs. 1 S. 3^1^ Gesetz über das Epidemiologische Krebsregister Niedersachsen (GEKN)). 3 mögliche Lösungen des Problems wurden diskutiert: (1) Das indirekte Linkage wird durch das niedersächsische Krebsregister durchgeführt. Dieser Ansatz konnte nicht umgesetzt werden, da er im Widerspruch zu den gesetzlichen Regelungen zur Nutzung von GePaRD steht, die eine weitere Überlieferung dieser Daten an Dritte vollständig ausschließen. (2) Es werden nur aggregierte Daten übermittelt. Dies hätte jedoch einen erheblichen Informationsverlust bedeutet und wurde deshalb nicht umgesetzt. (3) Das indirekte Linkage wird auf Basis einer 50 %igen Zufallsstichprobe des niedersächsischen Krebsregisters durchgeführt. Diese 3. Option wurde schließlich umgesetzt. Um abschätzen zu können, ob der angefragte Datenumfang für die Ziele der Studie ausreicht, musste zusätzlich eine Fallzahlschätzung der erwarteten Treffer beim indirekten Linkage durchgeführt und eingereicht werden.

### Schleswig-Holstein.

Obwohl zunächst geäußerte inhaltliche Bedenken geklärt werden konnten, wurde unser Antrag abgelehnt, da die derzeitige sich in der Überarbeitung befindende Landesverordnung (LVO) zur Durchführung von Kohortenstudien ein solches Linkage nicht vorsieht.

## Diskussion

In Deutschland wurde mit der Finanzierung der ersten neun Konsortien im Jahr 2020 mit dem Aufbau einer NFDI begonnen, um das Potenzial von Forschungsdaten im Sinne der FAIR-Prinzipien besser ausnutzen zu können. Allerdings ist es nicht ausreichend, nur Forschungsdaten in diesem Sinne zur Nachnutzung bereitzustellen. Es ist ebenso erforderlich, Gesundheitsdaten, die routinemäßig z. B. als Registerdaten, Abrechnungsdaten der Krankenkassen oder von Krankenhausinformationssystemen erfasst werden, für eine Nachnutzung zugänglich zu machen. Dabei ist zu erwarten, dass durch die personenbezogene Verknüpfung verschiedener Datenquellen eine größere Effizienz in der Nachnutzung von Daten im Gesundheitsbereich erreicht werden kann.

In diesem Zusammenhang sollte grundsätzlich versucht werden, verbindliche bundeseinheitliche Regelungen zu finden, da die verschiedenen länderspezifischen Auslegungen in vielerlei Hinsicht bundesweite Studien erschweren. Für die Daten aus klinisch-epidemiologischen Krebsregistern wurde in diesem Jahr vom Bundestag bereits ein neues Gesetz zur Zusammenführung von Krebsregisterdaten verabschiedet [26]. Dieses Gesetz, das am 18.08.2021 in Kraft getreten ist, wird zu einer Reihe von Änderungen führen, insbesondere sollen die Möglichkeiten der wissenschaftlichen Nutzung der Krebsregisterdaten für überregionale Forschungsprojekte verbessert werden. Dazu wird einerseits der Datensatz am ZfKD um Variablen der klinischen Krebsregistrierung ergänzt. Andererseits „ist die Schaffung eines kooperativen Datenverbundes mit projektbezogener Zusammenführung der Daten vorgesehen“ [27]. Darüber hinaus verfolgt die neue Gesetzgebung die Absicht, „die fallweise Verknüpfung mit anderen Datenquellen (z. B. Studien- oder Abrechnungsdaten) zu ermöglichen“ [27]. Zukünftig werden sich so hoffentlich deutlich verbesserte Bedingungen zur Nutzung der bundesweiten Daten ergeben, auch wenn damit keinesfalls sofort eine Außerkraftsetzung der landesrechtlichen Bestimmungen für die Übermittlung und Verknüpfung personenbezogener Daten ermöglicht wird.

## Fazit

Die Studie hat gezeigt, dass unter den derzeitigen Rahmenbedingungen die Verknüpfung von Registerdaten mit GKV-Abrechnungsdaten sehr aufwendig ist. Mit dem neuen Bundesgesetz zur Zusammenführung von Krebsregisterdaten werden in Deutschland Bedingungen geschaffen, die die Zusammenführung von personenbezogenen Gesundheitsdaten zu Forschungszwecken unter Einhaltung der Datenschutzbestimmungen befördern werden. Darüber hinaus sollte im Rahmen des Aufbaus einer NFDI neben der erforderlichen Infrastruktur und entsprechenden gesetzlichen Regelungen der Fokus besonders auf die Interoperabilität von Daten aus verschiedenen Quellen gelegt werden.

## Footnotes

1 „Dem Empfänger ist es verboten, die vom Krebsregister übermittelten Daten mit anderen Daten so zusammenzuführen, dass eine Identifizierung der betroffenen Person ermöglicht wird.“
